# New York County Medical Association

**Published:** 1905-01

**Authors:** 


### NEW YORK COUNTY MEDICAL ASSOCIATION.

"The Medical and Surgical Features of the Russo-Japanese War." Dr. L. L. Seaman read this paper. His remarks were practically the same as those made before the International Congress of Military Surgeons, held in St. Louis in October. His experience during the late Spanish war had taught him that the principal enemies of our army were ferment and microbes, and that the main fighting was done by the Medical Department. Led by the desire to see a conflict where powder and shell played at least an incidental part, he had visited the Japanese frontier. He had found the Military, Red Cross, and University Hospitals at Tokio conducted on broad, up-to-date principles. Up to July 1, only about 1,100 wounded had reached Tokio, and no medical cases. They were mainly from the Yalu, Nanshua, and Telissu fights, and included many severely wounded by shell fragments, bullets, shrapnel, and explosives. The physical condition of these patients was remarkable, considering the compaign through which they had passed and the severity of their wounds, for their faces showed little evidence of illness or suffering. Up to July 1st not one of these cases had ended fatally, and those remaining in the wards showed favorable prognoses. Little or no operating was done at the front, except in cases of extreme emergency, or when hemorrhage threatened death. All patients were treated by first-aid applications and were then sent to the rear as quickly as possible, and thence to the base hospitals in Japan. The Tokio surgeons complained that they had little to do, for by the time the soldiers arrived the vast majority of wounds had healed by first intention. At the base reserve hospitals at Hiroshima hundreds of wounded arrived every few days from the field around Port Arthur. The high velocity bullets, at such short range, produced almost every conceivable type of wound. The speaker had seen instances of bullets passing directly through the great cavities—seven through the cranial, nine through the thoracic, eight through the abdominal, and so many through the extremities that the number was quite lost, cauterizing their course and healing both entrance and exit wounds by first intention after first-aid dressings. Suppuration, even in cases in which the balls ricocheted, was comparatively rare. Operations for appendicitis, hernia, floating kidneys, gall-stones, etc., were conspicuous by their absence. The Japanese soldier had been taught how to treat his intestines. His diet was plain and rational, consisting of an easily prepared and easily digested ration, which could be thoroughly metabolized and converted into the health and energy that makes the Japanese soldier the ideal fighting machine of the world to-day. At Shimonoseki, the chief naval base, the character of cases in the hospital was of a type distinctly more severe. Prior to July 16th the total casualties in the navy amounted to 1,429, of which 1,209 were fatalities. Over 500 of these occurred on the occasion of the torpedoing of the Hatsuse, and a large proportion of the remainder were on the ships that were exploded or sunk. Less than 200 wounded were rescued, and of these only five had died, and the remainder were rapidly convalescing. The wounds in these cases were usually not from bullets, but from fragments of shell, causing fearful lacerations, contusions, comminuted fractures, etc. The medical wards of the hospitals were complete in every detail, but the beds were conspicuously empty, voicing more eloquently than words the most important lesson of the war. There were only a few cases of diseases of the respiratory tract, caused by unusual exposure, and scarcely a dozen under the head of diseases of the digestive system. Internal diseases were practically an insignificant factor in the naval hospitals, and up to July 20th not a single case of beriberi had developed. Dr. Takaki had demonstrated after an epidemic in 1882 that beriberi was a neurotic disorder, resulting from a lack of nitrogenous matter in the food. The ration was remedied and the disease practically eliminated from the hospitals of the Admiralty. The ration table contained a daily allowance of three ounces of liquor—saki—which was considered beneficial. A well-regulated army canteen existed, where beer was dispensed under official supervision, this beverage being recognized as bread in solution, that had undergone fermentation, thereby saving the stomach labor. He mentioned a number of medical officers in the Japanese army who had the rank and emoluments of major-generals, a rank at least two grades higher than the highest rank obtainable in the army of the United States by a surgeon. Dr. Seaman outlined the medical organization, showing how perfect the system was, how high the men ranked, and the duties that they performed. The Japanese were the first to recognize the true value of an army medical corps. Care of the sick and wounded consumed but a small part of their time. The solution of the greater problem, preserving the health and fighting value of the army in the field, by preventing disease, by careful supervision of the smallest details of subsisting, clothing, and sheltering the units, was their first and most important duty. The medical man was as much in the front as in the rear. He was with the first screen of scouts with his microscope and chemicals, testing water and foods, and investigating sanitary conditions. Notices were posted so that the approaching column was warned of danger where it existed. Bacteriological experts formed part of the staff of every divisional headquarters. A medical officer also accompanied foraging parties. In the camp he lectured the men on sanitation and personal hygiene. He had recognized that disease had brought more campaigns to disastrous terminations than the strategies of opposing generals or the bullets of their followers. Up to August 1, 9,862 patients, of whom 6,636 were wounded, were received at the Reserve Hospital at Hiroshima. Of these, up to that time, only thirty-four had died. This happy result was due to non-interference on the field and thorough antiseptic methods in after-treatment. In war four men died of disease for every one who fell from bullets. In this way the Japanese neutralized the superiority of Russian numbers. Japan was the first country in the world to recognize that the greatest enemy in war was not the army of the invader, but preventable disease. It was against this enemy that she had made her hardest fight and won her most signal victory. She had kept her men in superb condition. Major Seaman spoke of the contrast he had noted on visiting the Russian lines, where brutality, debauchery, and criminal carelessness existed. The three great lessons to be learned from the Japanese war were from the medical, the commissariat, and the transport department. The Japanese government permitted our government to send five military attaches to accompany their army in the field, but no surgeon or quartermaster had been detailed. The life-saving and life-preserving departments were omitted; the killing departments got the appointments, and the Japanese officers were laughing at our folly. But what could be expected of a government that, as in 1898–9, gave its men a ration that prostrated fifty per cent. of its 250,000 units with intestinal disease within six weeks and sent 3,000 to their last home? What could be said of the business principles of a Congress that preferred pensions to prevention; that permitted misguided women to deprive its army of one of its most beneficial features, a wellregulated canteen; that provided no instruction in physiology and hygiene in its great preparatory schools—Annapolis and West Point? Major Seaman said that it was time to demand another reorganization of our army, in which that branch of its service that grappled with the foe that caused eighty per cent. of its mortality should have equal recognition with other branches which, combined, fight the enemy which destroys but twenty per cent. The State deprived the soldier of his liberty, prescribed his dress, diet, exercise, etc., and expected him, if necessary, to lay down his life; it should therefore give him the best sanitation and the best medical supervision that the science of the age could devise. An adequate medical and sanitary organization was more important to the United States than to any other nation, because of the smallness of its regular army, and because in cases of emergency the vast majority of its medical officers must be drawn from civil life.

The Association of Military Surgeons had resolved that Congress be petitioned at its next session to reorganize the Medical Departments of the United States Army and Navy on a broad basis similar to that of the counties most advanced in military sanitation, giving to their officers equivalent rank, dignity and power, and to their personnel ample members for the proper care of the sick and injured. The Association also recommended that the sale of beer be permitted at army post exchanges, subject to such regulations as should be determined by the General Staff of the Secretary of War; that an adequate knowledge of the care of troops was of such vital importance that it should be given recognition in our Army and Navy Schools, and especially in the Staff Colleges and War Colleges, and that the present course at West Point and Annapolis should count it in the requirements for graduation; it therefore respectfully petitioned the President to make this resolution effective.

The speaker said that the subject was a terribly live one, and that all he hoped for was that the man in the ranks and the medical profession might get their rights and due recognition.

This paper was discussed by Major John L. Phillips, U. S. A., Dr. Nathan S. Jarvis, Captain A. E. Piorkowski, Imperial German Army, Colonel W. E. Church, U. S. A., Major J. F. Powell, U. S. A., and Dr. Andrew H. Smith, and was closed by Dr. Seaman.—*Medical Record.*

